# A controlled trial of value-based insurance design – The MHealthy: Focus on Diabetes (FOD) trial

**DOI:** 10.1186/1748-5908-4-19

**Published:** 2009-04-07

**Authors:** Alicen Spaulding, A Mark Fendrick, William H Herman, James G Stevenson, Dean G Smith, Michael E Chernew, Dawn M Parsons, Keith Bruhnsen, Allison B Rosen

**Affiliations:** 1 Department of Internal Medicine, University of Michigan School of Medicine, Ann Arbor, MI, USA; 2 Division of Epidemiology and Community Health, University of Minnesota School of Public Health, Minneapolis, MN, USA; 3 Department of Health Management and Policy, University of Michigan School of Public Health, Ann Arbor, MI, USA; 4 Department of Epidemiology, University of Michigan School of Public Health, Ann Arbor, MI, USA; 5 Department of Clinical, Social and Administrative Sciences, University of Michigan College of Pharmacy, Ann Arbor, MI, USA; 6 Department of Health Care Policy, Harvard University School of Medicine, Boston, MA; 7 University of Michigan Benefits Office, Ann Arbor, MI, USA; 8 Ann Arbor VA HSR&D Center of Excellence, Ann Arbor Veterans Affairs Medical Center, Ann Arbor, MI, USA

## Abstract

**Background:**

Diabetes affects over 20 million Americans, resulting in substantial morbidity, mortality, and costs. While medications are the cornerstone of secondary prevention, many evidence-based therapies are underutilized, and patients often cite out-of-pocket costs as the reason. Value-based insurance design (VBID) is a 'clinically sensitive' refinement to benefit design which links patient cost-sharing to therapy value; the more clinically beneficial (and valuable) a therapy is for a patient, the lower that patient's cost-sharing should be. We describe the design and implementation of MHealthy: Focus on Diabetes (FOD), a prospective, controlled trial of targeted co-payment reductions for high value, underutilized therapies for individuals with diabetes.

**Methods:**

The FOD trial includes 2,507 employees and dependents with diabetes insured by one large employer. Approximately 81% are enrolled in a single independent-practice association model health maintenance organization. The control group includes 8,637 patients with diabetes covered by other employers and enrolled in the same managed care organization. Both groups received written materials about the importance of adherence to secondary prevention therapies, while only the intervention group received targeted co-payment reductions for glycemic agents, antihypertensives, lipid-lowering agents, antidepressants, and diabetic eye exams. Primary outcomes include medication uptake and adherence. Secondary outcomes include health care utilization and expenditures. An interrupted time series, control group design will allow rigorous assessment of the intervention's impact, while controlling for unrelated temporal trends. Individual patient-level baseline data are presented.

**Discussion:**

To our knowledge, this is the first prospective controlled trial of co-payment reductions targeted to high-value services for high-risk patients. It will provide important information on feasibility of implementation and effectiveness of VBID in a real-world setting. This program has the potential for broad dissemination to other employers and insurers wishing to improve the value of their health care spending.

## Background

### Medication adherence in diabetes

As health care costs continue to increase, payers are searching for innovative interventions to strike a balance between containing costs and improving health outcomes, particularly for those with chronic conditions such as diabetes mellitus. An estimated 7% of Americans, representing 20.8 million people, have diabetes [1] and the prevalence continues to rise [2]. The micro- and macro-vascular complications of diabetes result in substantial morbidity and mortality [3] and contribute significantly to health care spending in the United States [4].

Medications are the cornerstone of secondary prevention for individuals with diabetes. Randomized controlled trials and national guidelines support the use of intensive glucose, blood pressure, and lipid management to reduce rates of serious complications and death [5]. Yet, medication adherence remains suboptimal [6,7]. In turn, poor adherence is associated with disease progression and complications, avoidable hospitalizations, increased health care costs, lost productivity, premature disability, and even increased mortality [8-12]. Further, it appears that depression, which often co-exists with diabetes, is associated with worse adherence, poorer health outcomes, and higher costs in individuals with diabetes [12-15].

### Impact of co-payments on adherence

While improving medication adherence is critical to improving diabetes outcomes, systematic reviews have shown little effect of informational, behavioral, and social interventions to improve adherence [16,17]. In contrast, a recent systematic review demonstrated the substantial impact co-payments have on medication adherence [18]. Cost sharing is generally applied in a non-targeted way, without regard to the medication's therapeutic benefit. Yet, this may create financial barriers to the very medications which would most benefit patients, raising two critical questions. First, can patients determine which drugs are most valuable to their own health? Second, will they choose to prioritize their medications accordingly to maximize health outcomes? Unfortunately, the evidence suggests that cost sharing indiscriminately reduces the use of both excess and essential (clear mortality and/or quality of life benefit) medications [18-24]. In turn, growing evidence suggests that individuals who decrease medication utilization due to cost have poorer health outcomes [18,21,22,24-27] and often incur higher health care costs [18,23,27].

### Value-based insurance design (VBID)

In response to growing evidence that 'one-size fits all' co-payments harm patients, a more nuanced approach to benefit design has been proposed. In this approach, co-payments are based on the expected clinical benefit from a drug, rather than solely on its acquisition cost [28-31]. Under such value-based insurance designs (VBID) the more beneficial the medication, the lower the co-payment. VBID effectively realigns the incentives faced by patients to increase utilization of and adherence to the most beneficial and valuable medications.

Interestingly, the appeal of VBID has taken root primarily outside of medicine – in the business world. In an effort to slow health care cost growth, Fortune 500 employer (and self-insurer) Pitney Bowes lowered co-payments for asthma and diabetes medications in 2001. While no rigorous evaluation was performed, they reported a one-year one-million dollar savings to the Wall Street Journal [32]. Following Pitney Bowes' lead, several other employers (*e.g*., Marriott, Procter & Gamble, Florida Power and Light) have implemented VBID programs. Employer benefit consultants (*e.g*., Hewitt, Mercer), disease management companies (*e.g*., ActiveHealth Management), pharmacy benefit managers (*e.g*., SXC Health Solutions, Prime Therapeutics) and health plans (*e.g*., Aetna) have also launched VBID-related products. While these benefit designs hold promise for improving value of care, there have been few controlled evaluations, and none that target co-payment reductions to specific services for specific patient populations. Yet, 'targeting' is critical to improving health care value. Patient risk and, therefore, benefit from health services, is heterogeneous, with most services providing significantly higher value for patients at highest risk. By altering co-payments so that the strongest incentive to take a medication is targeted to those who will most benefit from that therapy, the more likely the system will be to maximize the health returns to spending, thereby maximizing value.

## Impetus for intervention trial

### Institutional environment

In 2004, amidst pervasive gaps in both quality and access to health care and continually increasing health care costs (particularly for employers in Michigan), the University of Michigan (UM) announced a major initiative to develop, implement, and evaluate new models of care to improve the health and well-being of the UM workforce in a cost-effective manner. As part of this initiative, the MHealthy: Focus on Diabetes (FOD) trial was designed and implemented as a targeted co-payment reduction intervention for UM employees and their dependents with diabetes. The intervention was designed as a prospective controlled study to allow for rigorous evaluation of the program's impact.

### Needs assessment

Diabetes was chosen for the focus of this intervention following a needs assessment which identified diabetes as prevalent and adherence to evidence-based pharmacotherapies as suboptimal in the UM population. While the average patient with diabetes requires multiple medications for adequate glycemic control [5], over half the UM employees and dependents with diabetes were using only one hypoglycemic agent, suggesting a potential opportunity for improvement. In turn, fewer than half were on an angiotensin converting enzyme inhibitor or angiotensin receptor blocker (ACE/ARB), and only half were on HMG-CoA reductase inhibitors (statins). The critical role of co-payments was documented in a prior investigation in M-CARE (the setting of the current study), in which Ellis and colleagues found two-year statin discontinuation rates of 50% to 100%, with the highest co-payments associated with a four-fold increase in discontinuation [19].

### Therapies selected for co-payment reductions

Interventions were selected based on evidence of health benefits, guideline indications for use [5], and documented underutilization in clinical practice. They included statins, ACE/ARBs, other antihypertensives, and all hypoglycemic agents. Co-payments were also reduced for antidepressants, as evidence suggests that diabetes self-management practices (including medication adherence) are better when comorbid depression is adequately treated [33].

## Methods

### Study overview

The aim of this study was to examine the impact of targeted co-payment reductions (targeted to high-value but underutilized services) for an employed population with diabetes. The intervention, initiated on 1 July 2006, comprised two elements: an educational letter detailing the importance of medication adherence in diabetes, and targeted co-payment reductions for several high-value therapies. The intervention group received both elements (detailed below), while a control group received the educational letter alone.

### Hypotheses

We hypothesized that the removal of financial barriers to evidence-based, high-value therapies will result in improved uptake and adherence, and a more efficient use of resources. Specific hypotheses are:

1. Compared to individuals with diabetes with usual co-pays, those receiving targeted co-payment reductions for high-value therapies will increase uptake of these therapies and improve adherence to these therapies (conditional upon their use).

2. Compared to payers for individuals with diabetes with usual co-pays, payers for those receiving value-based co-payment reductions will incur higher pharmaceutical spending, lower non-pharmaceutical cost growth, and lower overall cost growth. Importantly, we do not hypothesize an absolute financial savings over previous years. Rather, we posit that the rate of non-pharmaceutical cost growth will be slower in the intervention group than in the controls.

3. Among individuals with diabetes and depression, compared to individuals with usual co-pays, individuals receiving value-based co-payment reductions for high-value therapies will have improved uptake and adherence to antidepressants and improved uptake and adherence to other study medications (statins, ACE/ARBs, other antihypertensives and glycemic agents).

### Study setting

The University of Michigan is a large Midwestern university with an enrollment of nearly 50,000 students. The university contracts with a single pharmacy benefits manager (PBM), SXC Health Solutions, for all of its employees, dependents, and retirees. Retirees were excluded from this study, however, because Medicare Part D and the FOD intervention were implemented in close proximity, making it difficult to isolate the effects of each from the other. The approximately 70,000 active UM employees and dependents are enrolled in several different health plans. The most frequently chosen plan is M-CARE, which, in 2006, enrolled approximately 81% of active UM employees and dependents.

M-CARE is a UM-owned non-profit independent-practice association model health maintenance organization (HMO) with approximately 200,000 enrollees throughout Southeastern Michigan in 2006. While UM enrollees use the university-contracted PBM (SXC Health Solutions), all other M-CARE enrollees receive pharmacy services through CatalystRx, the M-CARE-contracted PBM at the time the FOD intervention was initiated. Both SXC and CatalystRx use the same claims processing platform, allowing for acquisition of comparable data for both the intervention and control groups in this study.

M-CARE has a diabetes disease management program designed to improve patient adherence to recommended diabetes care processes. Therefore, any measured effect of targeted co-payment reductions is actually the incremental additional effect beyond the impact of the disease management program.

### Study population identification

The initial intervention population included 2,507 UM employees and dependents enrolled in UM's pharmacy benefits plan and identified as individuals with diabetes based on at least one pharmacy claim for a diabetic hypoglycemic medication (oral, injectable, or inhaled) within the 12 months prior to intervention.

**Figure 1:**
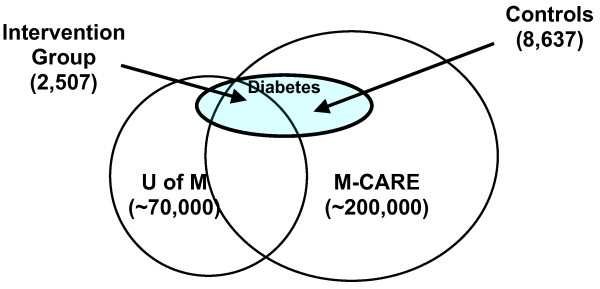
**Identification of intervention and control groups**. The large circle on the left (labeled 'U of M') depicts University of Michigan employees and dependants. The large circle on the right (labeled M-CARE) depicts M-CARE enrollees, with the central area of overlap representing UM employees enrolled in M-CARE. The subset of individuals with diabetes is depicted by the shaded circle labeled 'Diabetes.' The intervention group includes UM employees and dependents with at least one pharmacy claim for a glycemic medication (oral, injectable, or inhaled) within the 12 months prior to the study timeframe. The control group consists of M-CARE enrollees who are employees and dependents of other, non-UM employers with at least one pharmacy claim for a glycemic medication within the 12 months prior to the study timeframe.

The initial control population included 8,637 M-CARE enrollees receiving coverage through other employers and identified as individuals with diabetes using the same pharmacy claims criteria. Figure 1 depicts the intervention and control groups and their relationship to the University and M-CARE.

While UM employee turnover is low, the population eligible for the intervention is not static. Both new UM employees with pre-existing diabetes and current employees newly diagnosed with diabetes are automatically enrolled in the intervention upon having an eligible pharmaceutical claim filed. The automatic enrollment design was chosen to reduce the burden of action required for individuals to benefit from the co-payment reductions. Participants could choose to opt-out of the program at any time. Further, UM employees and dependents not identified from pharmacy claims can opt-in to the program by contacting the Focus on Diabetes program coordinator and self-identifying that they have diabetes. While eligible for the full intervention, these 'opt-in' individuals will be excluded from analyses because there is no comparable control group.

### Approvals and data safety and monitoring

The study was approved by the University of Michigan Institutional Review Board and the Research Review Committee of M-CARE. All data are obtained and managed by the UM pharmacy benefits manager, SXC Health Solutions, and by M-CARE, both of which require personal health information (PHI) in the course of their usual activities. To ensure protection of confidentiality, unique study identifiers are assigned to each patient and data are stripped of PHI prior to transmission to the evaluation team. Because the intervention constitutes a quality improvement initiative (taking place regardless of evaluation) and data are provided in limited datasets, informed consent requirements were waived. However, individuals were given the option to opt-out of the intervention at any time.

Because the intervention involved a benefit change, additional approvals were obtained from the three unions representing UM employees.

### Study design

The study employs an interrupted time series design, with medication adherence rates assessed at three-month intervals (unit of observation is the patient-quarter), beginning 30 months prior to the intervention (the 'pre' period) and continuing through the duration of the 30-month intervention (the 'post' period). This study design provides a strong test of the intervention's impact on medication adherence, separate from other ongoing temporal changes in adherence [34]. The advantage of this 'difference-in-difference' design is that any change in the control group values may reflect naturally occurring changes over time (perhaps due to policy or medical care changes), while any change in the UM intervention group values will reflect both the same naturally occurring trends, as well as the impact of the value-based co-payment reductions.

## Procedures

### Intervention

#### Educational letter

One month prior to the co-payment reductions, an educational letter was sent out, with information on the general health benefits of medication adherence and the specific benefits of ACE/ARBs, statins, and tight glycemic and blood pressure control in diabetes. A phone number for a nurse case manager was provided for questions. A brief description of the impending co-payment reductions was provided, as well as a statement allowing individuals to opt-out of the study. A parallel educational letter was sent to the control group, differing from the UM letter only in exclusion of information about the co-payment reductions. The control group was also provided with a phone number for a nurse case manager available for questions [35,36].

#### Co-payment reductions

While the intervention was designed to reduce financial barriers to effective therapies, it was not meant to preclude the concomitant use of other incentive structures already in place. When the intervention went into effect, UM had a three-tiered formulary with co-pays of $7, $14, and $24 for generic (tier one), preferred brand (tier two), and non-preferred brand (tier three) medications, respectively. This underlying benefit structure was left intact with the value-based benefit laid on top. To maintain the underlying incentives to use lower cost drugs within a class, the intervention lowered co-pays in a graded fashion (tier one co-pays decreased by 100%, tier two by 50% and tier three by 25%) to a new three-tiered benefit structure of $0, $7, and $18 co-payments, respectively.

#### Data collection

Pharmacy claims were obtained for the intervention group from SXC Health Solutions, and for the control group from CatalystRx. Non-pharmacy claims were obtained from M-CARE for both groups. All data were transferred to the M-CARE claims administration office, which assigned unique study identifiers, managed the linkage of patient data across data sources and over time, and stripped the data of PHI prior to transfer to the evaluation team.

#### Measures

##### Primary outcomes

The primary outcomes include medication utilization and medication adherence. We define medication utilization (or uptake) as at least one pharmacy fill of a medication in the drug class of interest during each one-year time window (two pre- and two post-intervention); this is analogous to the calculation of many performance measures, including several Healthcare Effectiveness Data and Information Set (HEDIS) measures [37]. For statins and ACE/ARBs, medication uptake rates will be explored in the subset of individuals identified by claims or laboratory data to have a clear clinical indication for use (*e.g*., statins for individuals with diagnosed hyperlipidemia or LDL cholesterol above guideline recommended levels [5]).

We define medication adherence using medication possession ratios (MPRs). The MPR is the ratio of the cumulative days of medication supply obtained, divided by the number of days supply which would be needed for perfect adherence. To calculate the MPR, each day in a quarter will be evaluated as 'covered' or 'not covered' by a refill; if all days are 'covered' by a refill then the MPR will be 100%. MPRs will be calculated separately for each medication class (listed in Table 1) for each quarter. Rules for handling early refills, dosage increases, and within-class drug switches will be applied and have previously been described [38]. For hospitalizations, we assume medications are hospital-supplied until discharge, at which time the home supply is resumed. While the MPR is a measure of refill adherence rather than a direct measure of medication taking behavior, it has been shown to correlate well with patient outcomes [39].

**Table 1:** Drug classes receiving co-payment reductions: focus on diabetes trial

**Glycemic Agents**
Metformin
Sulfonylureas
Thiazolidinediones
All other glycemic agents (except insulin*)
**Antihypertensive Agents**
ACE-Inhibitors & Angiotensin Receptor Blockers (ACE/ARB)
Beta blockers
Calcium Channel Blockers
Diuretics
Other antihypertensives
**Lipid-Lowering Agents**
HMG-CoA Reductase Inhibitors (statins)
Zetia
Other lipid lowering agents
**Antidepressants**
SSRSs/SNRIs
Tricyclic agents
Other antidepressants

*At the time of the intervention, M-CARE already waived copayments for Insulin.

##### Secondary outcomes

Secondary outcomes include health care utilization rates (*e.g*., counts of outpatient visits, ER visits, hospitalizations) and health care spending. Pharmaceutical and non-pharmaceutical expenditures will be examined both overall and disaggregated into the costs borne by the payer and those assumed by the patient (*i.e*., out-of-pocket costs).

##### Model covariates

To ensure findings are due to the co-payment reductions and not to underlying differences between the two groups, models will adjust for age, gender, comorbidity, member status (employee, spouse or dependent), and number of medications taken. Comorbidity is assessed using the Deyo modification to the Charlson comorbidity index [40], a well-validated measure of the burden of comorbid illness that was developed specifically for use with claims data.

##### Statistical power

The intervention can increase appropriate medication use in two ways: by increasing use in those currently not on the medications of interest (*i.e*., increasing uptake), and by increasing the adherence of patients who are taking the medications. Based upon our original estimated sample sizes of 2,131 in the intervention group and 4,000 in the control group, and an average initial uptake rate of 50% (based upon statins and ACE/ARBs), we will have 80% power to detect an effect of 3.8% change in medication utilization at a p = 0.05 significance level. In turn, based upon average pre-intervention medication adherence rates of 65% (based on statins and ACE/ARBs), we will have 80% power to detect an effect of 3.5% change in MPR (*i.e*., adherence) at the p = 0.05 significance level.

### Statistical analyses

#### Medication utilization (or uptake)

We classify an individual as utilizing therapy if any of their dispensed medications include a drug within the drug class of interest, either alone or in combination with another drug during each of the two one year windows in the pre-period and two one-year windows in the post-period. For analysis purposes, ACE-Inhibitors and ARBs are considered one drug class.

#### Medication adherence

**Figure 2:**
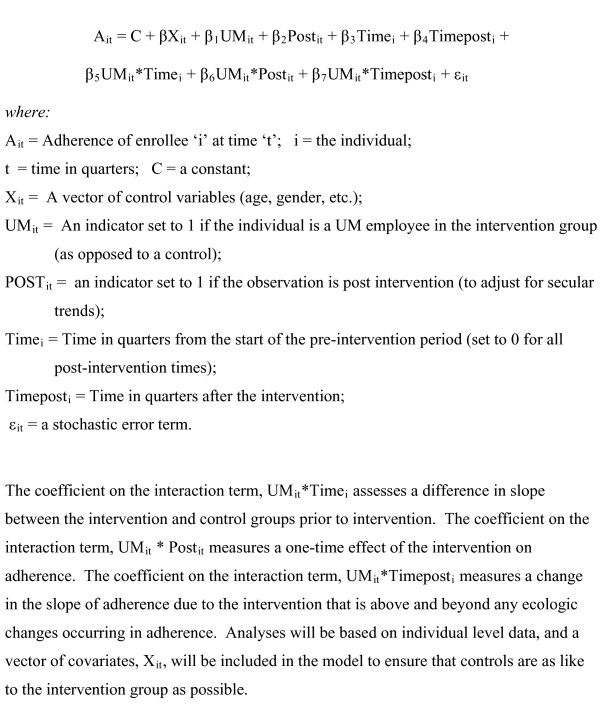
**Segmented multiple time series regression model**.

Analyses will assess both for a one-time effect of the intervention on adherence and for a change in the rate of change in adherence over time. Appropriate statistical adjustments will be made to account for multiple observations (at each three-month interval) on the same individuals. Our basic model will be a segmented multiple time series regression, as specified in figure 2.

#### Health care expenditures

To implement the 'difference in difference' approach to examining health care costs, we will use the same multiple time-series regression approach specified above. Initial cost models will use ordinary least squares with a log transformation of costs to account for the skewness of the distribution of health care expenditures. We will test two-part econometric models against the one-part model because the unit of analysis is the patient quarter, and there may be several individuals with no spending in any given quarter. The first part model will be a logit/probit modeling the probability of non-zero expenditure, and the second part will model expenditure conditional on non-zero spending [41].

#### Subgroup analyses

Because depression is associated with worse adherence and higher costs in individuals with diabetes [12-15], subgroup analyses will be performed to explore for a differential effect of the intervention in those with depression. In turn, because there may be important differences between UM employees who enroll in M-CARE and those who enroll in other health plans, analyses will be repeated with and without the small subset enrolled in other health plans.

## Results

### Sample characteristics

Over the first year, a total of 2,507 UM employees and dependents with diabetes were included in the intervention program, while 8,637 employees with diabetes and dependents with diabetes of other employers were identified for the control group. Table 2 shows the baseline characteristics of both groups. The mean age was 45.1 years in the intervention group and 47.5 years in the control group. The intervention group had slightly more women than the control group (57.7% versus 53.1%), but health was comparable between the two groups, with Charlson scores of 1.43 and 1.46 in the intervention and control groups, respectively. The baseline rates of metformin and statin use were similar between the two groups. In contrast, baseline SSRI use was significantly higher (21.7% versus 18.9%), and ACE/ARB use significantly lower (48.7% versus 43.4%, P < 0.01) in the intervention group relative to the controls.

**Table 2:** Baseline characteristics of the MHealthy: focus on diabetes sample

**Characteristics**	**Reduced Co-payments (Intervention)**	**Standard Co-payments (Control)**
**Number of respondents enrolled**	2,507	8,637

**Mean age in years (SD)**	45.1 (13.06)	47.5 (12.83)*

**Female**	57.7%	53.1%*

**Basis for insurance coverage**		
**Employee**	62.3%	66.3%*
**Spouse**	31.1%	29.1%
**Child**	4.7%	4.0%
**Other**	1.9%	0.6%

**Median Household Income**^†^	$55,086	$54,758

**Charlson Comorbidity Score**^‡^**(SD)**	1.43 (0.65)	1.46 (0.69)

**Baseline Medication Utilization**		
**Metformin**	53.9%	53.6%
**ACE/ARBs**	43.4%	48.7%*
**Statins**	44.7%	44.5%
**SSRIs**	21.7%	18.9%*

*Significant at P < 0.05

^†^Based-upon zip-code level median household income data

^‡^Estimates based on the Deyo modification to the Charlson Index [40].

### Acceptability of intervention

The FOD Trial was successfully implemented on 1 July 2006. Comprehensive data is currently being collected and will be fully analyzed upon completion of the 30-month intervention window. The automatic enrollment design was well-received, with very few individuals choosing to opt-out of the program. Numerous e-mail testimonials have been received from employees and dependents that have benefited from the program. In addition, the intervention has been very well-received by various stakeholders including those at the University of Michigan [35,42] and the Michigan state legislature [43], who have expressed interest in expanding such programs to other chronic diseases, pending the results of this trial.

## Discussion

As health care costs continue to rise, payers are increasingly shifting costs to patients through higher deductibles and co-payments [32]. Yet, uniform increases in co-payments may curb the use of both low-value and high-value therapies alike, potentially resulting in adverse health consequences in individuals with chronic diseases [18,44]. VBID has been proposed specifically to offset the adverse clinical effects of rising out-of-pocket costs, by setting the patient co-payment amount relative to the value – not exclusively the cost – of the therapy [28-31]. While several large employers and insurers are experimenting with VBID type programs, there have been no prospective controlled evaluations of the impact of such programs in actual practice.

This trial is the first of its kind – an intervention of targeted co-payment reductions for specific high-value therapies in specific high-risk patients – designed both to improve adherence to evidence-based therapies by patients with diabetes in the workforce, but also to allow for rigorous evaluation of the program's effectiveness. The trial's progress to date has demonstrated the feasibility of implementing targeted, value-based benefit design changes in practice. The automatic opt-in design of the intervention has created near unanimous participation. No significant barriers to implementation have been encountered to date, and the intervention continues to receive strong employee-level and institutional-level support at UM. Planned analyses will determine the intervention's impact on the uptake of and adherence to evidence-based medications, as well as the impact on the use and cost of both pharmaceutical and non-pharmaceutical health services.

This study has some limitations. First, determining the effect of the co-payment reductions above any effects due to secular trends in adherence can be difficult. Second, although the control group was identified from the same health plan using the same selection criteria used to identify the intervention group, differences remain between these two groups because randomization was not part of the study design. That noted, the interrupted time series, control group design was specifically chosen because it provides a strong test of whether the copayment reductions impact on medication adherence, separate from other ongoing temporal changes in adherence or baseline differences between the two groups [34].

Third, this trial is taking place in an evolving health care marketplace, which may make it more difficult to isolate the effect of the intervention from other ongoing benefit design changes or quality-improvement initiatives. Again, FOD's interrupted time series control group design offsets this concern, to the extent possible, and is a key methodological strength of the trial. By assessing outcomes for an intervention and a control group at multiple times both before and after the intervention, we capture and can control for temporal trends due to other market factors, provided they do not occur in only one of the two groups at the same time as the intervention is implemented.

Fourth, adherence is estimated using medication possession ratios, which assume that the supply of medication dispensed is an adequate proxy for patient adherence. However, because the intervention should have a direct effect on pill-buying but only an indirect effect on pill-taking, MPR is likely to modestly overestimate actual adherence, by causing some unknown number of patients to buy but not take their medications. Further, if physicians instruct patients to take medications differently than prescribed, or if medications are stockpiled or conversely lost, MPR may be an imperfect measure. However, the use of MPR as a measure of adherence for controlled trials has been validated by Steiner and colleagues and is used extensively in clinical research [45]. Fifth, the trial focuses on individuals with diabetes with employer-based health insurance. While over 62% of the non-elderly U.S. population has employer-based coverage [46], there is tremendous heterogeneity in employer benefit packages and in the health risk profiles of employees, potentially limiting generalizability.

Sixth, a common difficulty with any policy intervention is assessing for unintended consequences (or externalities) which may arise as a result of the intervention. To the extent we have anticipated them, we will examine for potential externalities, such as formulary tier-shifting, in which individuals shift from lower to higher tier drugs in response to the co-payment reductions. Our intervention maintained a cost advantage for using lower tiers, but the extent of tier-shifting remains an empirical issue that we will explore.

Finally, a practical issue related to the likely uptake of VBID-type interventions merits mention. With health care costs rising rapidly, payers are looking for ways to constrain cost growth, potentially limiting the appeal of benefit design alterations which shift pharmaceutical costs back to the employer from the patient. For the most part, employers want to see a positive return on investment, which is unlikely when framed solely in financial terms. However, the true return on investment in health care is health. When framed in these terms, employers should expect and even demand a positive return on investment.

In conclusion, we have described the rationale, design, and implementation of a prospective controlled trial designed to test the effectiveness of targeted, value-based co-payment reductions in improving medication adherence among a population of individuals with diabetes covered by employer-sponsored health insurance. Findings from this study should provide needed insight into the responsiveness of patients' medication-taking behaviors to targeted reductions in out-of-pocket costs, as well as the impact on both pharmaceutical and overall health care utilization and spending. This study is quite timely given rapidly rising health care costs, increasing use of consumer-directed health care (with its high cost-sharing requirements), and continuing pervasive quality gaps between the optimal use of high-value chronic disease therapies and their actual use in practice [47,48]. Data from this study will provide payers with needed insights into the role of VBID to mitigate the adverse health consequences of underuse due to high out-of-pocket expenditures, while continuing to use cost-sharing to discourage overuse.

## Competing interests

The authors declare that they have no competing interests.

## Authors' contributions

ABR had full access to all study data and takes responsibility for the integrity of the data and accuracy of the data analysis. AS, AMF, WHH, JGS, DGS and ABR were responsible for study concept and design. KB and DP acquired the data. AMF, WHH, JGS, DGS, MEC and ABR analyzed and interpreted the data. AS, AMF, WHH, MEC and ABR drafted the manuscript. JGS, DGS, and KB critically reviewed the manuscript for important intellectual content. ABR and AMF supervised the study. WHH, DP and KB offered administrative, technical, or logistic support.
